# Supplementary material for: Personal Genome Project UK (PGP-UK): a research and citizen science hybrid project in support of personalized medicine
Source: BMC Med Genomics. 2018 Nov 27;11:108. doi: 10.1186/s12920-018-0423-1 (PMC6257975; doi:10.1186/s12920-018-0423-1)
Supplement: Supplementary file 1 — Exemplar PGP-UK genome report of participant uk35C650 providing details on the number and type of variants and their association with ancestry as well as possibly beneficial and harmful traits. The released versions of the reports provide links to the underlying databases, including SNPedia, gnomAD, GetEvidence and ClinVar. (PDF 203 kb) [file 12920_2018_423_MOESM1_ESM.pdf]

PGP-UK Genomics Report for PGP-UK1/uk35C650

1 Summary

This is the genome report was produced using collaborative research tools, including SNPedia and GetEvidence. This section shows an overview of all the small variants which were found in the genome for this individual, when compared with a reference genome. These variants are summarised in Table 1 and the pie-charts in Figures 2, 3 and 4.

This report was generated automatically and is not clinically approved. It is provided for personal and research purposes only.

This document contains hyperlinks, shown in grey, that will take you to external websites where you can find more detailed explanations. Some of the technical terms are also explained in more detail in the Ensembl Glossary. We would welcome your feedback about this report, for example, if you would like more information about anything or if any of the links have become inactive. You can contact us on: pgp-uk@ucl.ac.uk.

This summary shows an overview of all the variants which were found in the genome for this individual. The "variants remaining after filtering" refers to any differences in the DNA identified when compared to the reference genome. Of these, the majority will have already been found in some other sequenced individual and put on a database (existing variants) while others have not yet been annotated (novel variants).

"Overlapped genes" refers to the number of times where a variant was found in a region of the genome containing a gene. The diagram in Figure 1 is a simplification of the usual gene structure. "Exon" refers to the part of the gene which goes on to form a protein, and variants in this part of the gene are more likely to cause changes in the shape of the protein. Upstream, downstream, intronic and intergenic variants are more likely to alter the regulation of that gene but will not change the protein itself.

A transcript for a protein-coding gene can include the exons, introns and other gene features that are transcribed and important for gene function but might not be translated into the final protein. Not all transcripts are for protein-coding genes, with many containing non-coding RNAs that can be overlapping other genes, in introns or in intergenic regions.

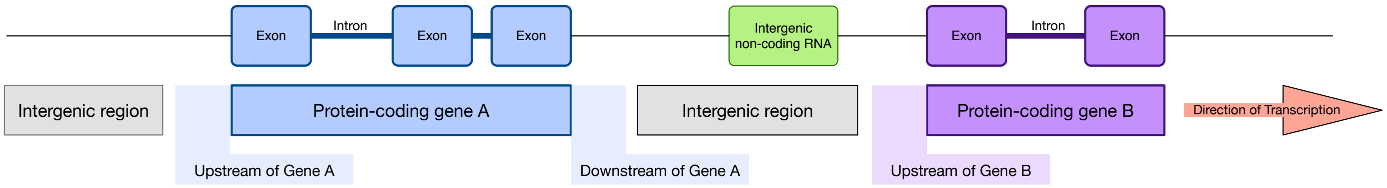

Figure 1: Diagram of gene structure indicating locations of potential variants

Feature	Count
Lines of input read	4130989
Variants remaining after filtering	4105273
Novel / existing variants	103667 (2.5%) / 4001606 (97.5%)
Overlapped genes	54674
Overlapped transcripts	64349
Overlapped regulatory features	211555

Table 1: Variant calling summary

There are several different types of genomic variants. The most common change is when one single building block of the DNA (called a nucleotide) is changed, called a single nucleotide variants (SNV). Other variant types include insertions, where the DNA in the individual is longer than the reference sequence due to the insertion of one or more nucleotides; and deletions, where a few nucleotides are missing compared to the reference sequence.

Some of these changes will have no effect on the protein, while some changes may alter the protein function to varying degrees. The PolyPhen analysis software attempts to quantify the effect each mutation will have on the protein function. This ranges from "benign" where no change to the protein function is expected, to "probably damaging" where it is predicted that the mutation will affect protein function. It is nevertheless important to note that what is "damaging" for the protein is not necessarily damaging for the individual.

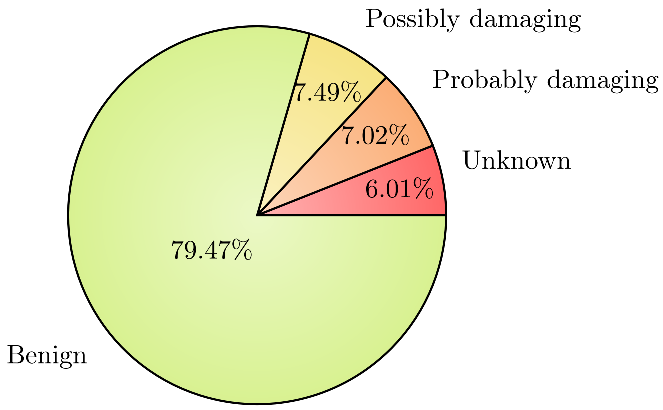

Figure 2: PolyPhen Summary

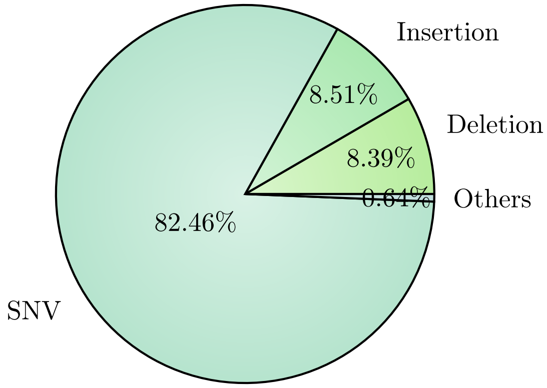

Figure 3: Variant Class

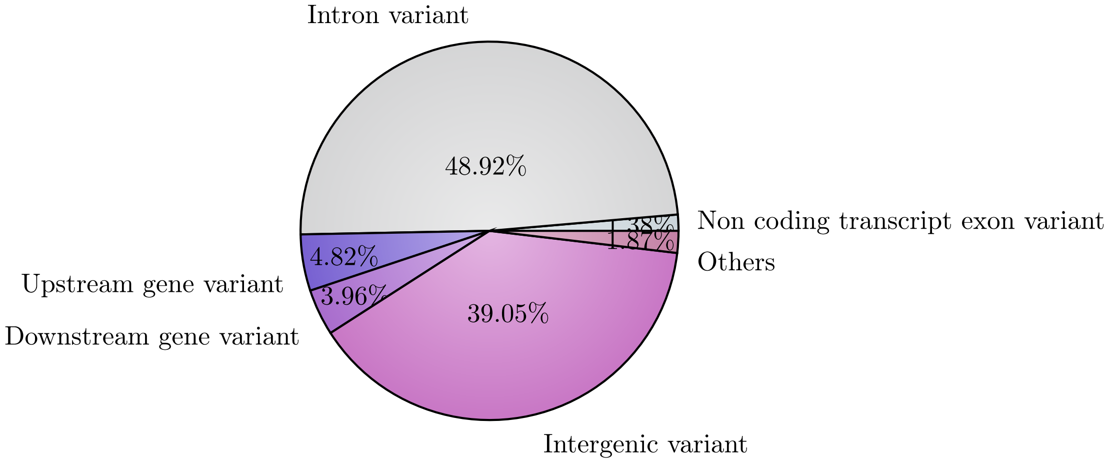

Figure 4: Consequence type

2 Ancestry

This plot shows the distribution of the genomes of different populations. Data from several studies which used whole genome sequencing was used to see the relationships between the genomes of the populations. It shows how closely related certain populations are genetically: Groups which cluster closely are more genetically similar than groups which are further apart. The black star symbol shows where this PGP-UK participant sits in relation to other populations, indicating their ancestry and their most closely related populations according to genetic sequence.

Please note that this analysis is limited by the populations available in the 1kGP data. If there are European subpopulations reported, and the ancestry of the participant does not correspond to any of the 1kGP populations, the closest 1kGP sampled subpopulation will be shown (even though it might be different from the participant's actual ancestry).

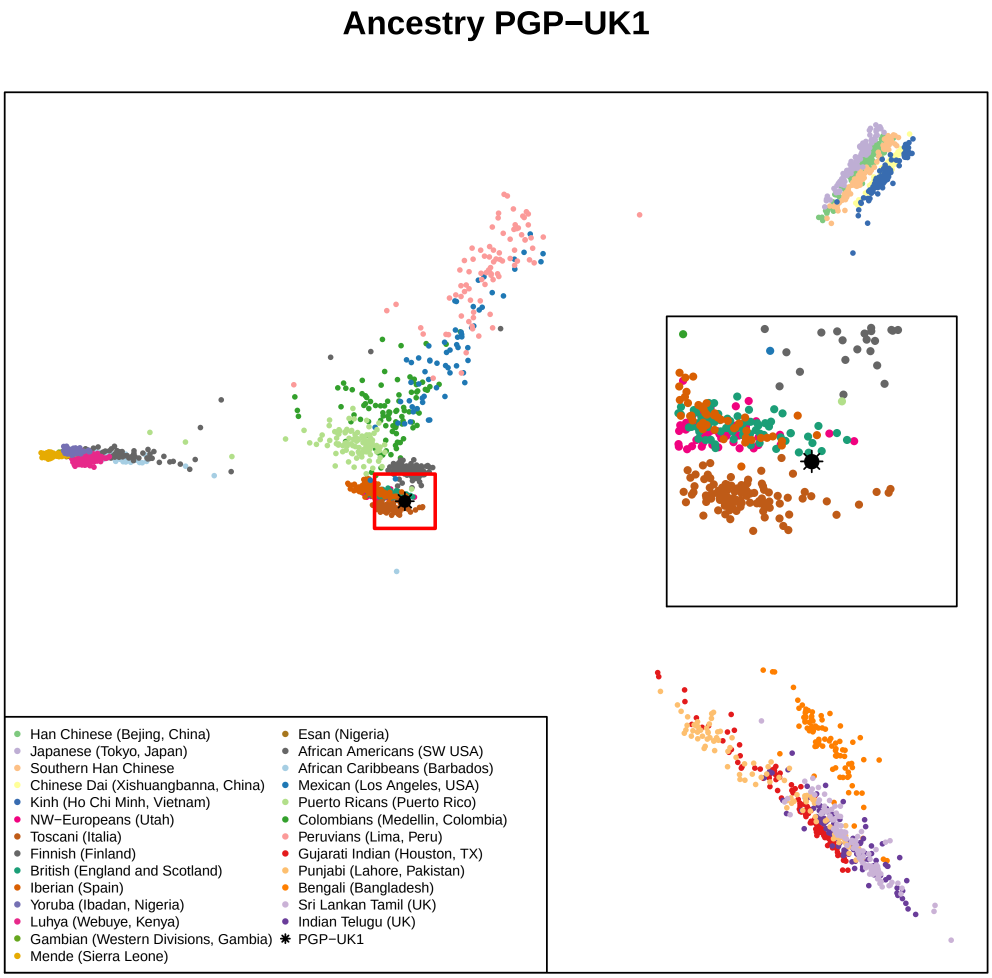

Figure 5: Ancestry Principal Component Analysis

3 Traits (based on SNPedia information)

Existing research has associated many variants with phenotypic traits, some of which can be perceived as beneficial while others appear to have a harmful effect. Some traits are complex and can be affected by several variants. It is likely that some of these would confer a higher risk while others a lower risk of trait manifestation. These can not be combined linearly to produce an actual risk of disease.

It is important to note that in most cases genomic data is probabilistic, not deterministic- i.e. having a genetic predisposition for a disease is not a diagnosis; rather, it shows an increased likelihood of developing that disease. Also, one person can have both potentially beneficial and harmful variants in the same gene, or associated with the same disease.

Some variants can also affect certain populations more, or will only affect a particular gender. For example, a variant for higher risk of endometriosis in the sequence of a male will not directly affect that person, but can be passed on to descendants.

While many traits are the result of a unique variant, many are the combination of several variants throughout the genome. In SNPedia, these are called genosets. These can integrate some of the information already present in the single variant tables, or be the combination of variants that have no phenotypic effect on their own, but contribute to a trait when together.

The variants in the following tables are sorted by magnitude. This is an subjective measure defined in SNPedia to highlight the perceived importance of the genotype described. At the moment this scale goes from 0 to 10. You can read more about it by visiting their explanatory webpage.

As our knowledge grows, the interpretation of the effect of certain variants might change. Clicking on the links in the genome report tables will take you to websites containing more information about each variant.

3.1 Possibly Beneficial Traits

Mag.	Identifier	Genotype	Summary	GnomAD	GetEvidence	ClinVar
3	rs7294919	(C;T)	Moderately enhanced hippocampal volume	Link		
3	rs8177374	(C;T)	Resistance to several diseases	Link	Link	Link
2.5	rs2943634	(A;A)	Lower risk of ischemic stroke	Link	Link	
2.2	rs2511989	(A;A)	0.44x decreased age-related macular degeneratio...	Link	Link	
2.1	rs547154	(A;A)	0.47x decreased risk for AMD	Link		Link
2.1	rs806380	(G;G)	Uncommon. lowest odds of cannabis dependence	Link		
2	rs1026732	(A;A)	<0.70x risk for restless legs	Link	Link	
2	rs10503669	(A;C)	Associated with higher HDL cholesterol	Link	Link	
2	rs10504861	(A;G)	Reduced risk of migraine without aura	Link		
2	rs11635424	(A;A)	<0.70x risk for restless legs	Link	Link	
2	rs12593813	(A;A)	<0.71x risk for restless legs	Link	Link	
2	rs12678919	(A;G)	Associated with higher HDL cholesterol	Link	Link	
2	rs12979860	(C;C)	~80% of such hepatitis C patients respond to tr...	Link	Link	Link
2	rs1544410	(G;G)	Decreased risk of low bone mineral density diso...	Link	Link	
2	rs1799884	(G;G)	Mothers have typical Birth-Weight babies. Sligh...	Link		
2	rs1800972	(G;G)	Reduced risk for Crohn's disease; reduced risk ...	Link		
2	rs1864163	(A;G)	Associated with higher HDL cholesterol	Link	Link	
2	rs2060793	(A;A)	Lower serum levels of vitamin D	Link		
2	rs2235015	(G;T)	Somewhat more likely to respond to certain anti...	Link	Link	
2	rs261332	(A;G)	Associated with higher HDL cholesterol	Link		
2	rs3738579	(C;T)	0.5x decreased risk for cervical cancer: HNSCC:...	Link		
2	rs3764261	(G;T)	Associated with higher HDL cholesterol	Link	Link	Link
2	rs3914132	(C;T)	Lower otosclerosis risk	Link	Link	
2	rs4143094	(G;G)	No increased risk of colorectal cancer correlat...	Link		
2	rs4149268	(G;G)	Associated with higher HDL cholesterol	Link	Link	
2	rs4585	(G;G)	Slightly higher (1.35x) odds of good metformin ...	Link		Link
2	rs505922	(T;T)	Blood type O	Link	Link	

Mag.	Identifier	Genotype	Summary	GnomAD	GetEvidence	ClinVar
2	rs6505162	(A;C)	0.58x decreased risk for esophageal cancer	Link		
2	rs6511720	(G;T)	Slightly lower odds of developing CHD.	Link	Link	Link
2	rs7776725	(T;T)	Stronger bones	Link	Link	
2	rs801114	(T;T)	0.78x decreased Basal Cell Carcinoma risk.	Link	Link	
1.8	rs1800588	(C;T)	Higher HDL-C levels	Link	Link	
1.8	rs187238	(C;G)	Hypertension not a risk factor for sudden cardi...	Link		
1.8	rs4714156	(C;C)	<0.61x risk for restless legs	Link		
1.8	rs6897932	(C;T)	0.91x decreased risk for multiple sclerosis	Link	Link	Link
1.5	rs11136000	(C;T)	0.84x decreased risk for Alzheimer's disease	Link	Link	
1.5	rs11212617	(C;C)	Somewhat increased likelihood of treatment succ...	Link		Link
1.5	rs309375	(G;G)	Smaller mosquito bites	Link		
1.5	rs3851179	(A;A)	0.85x decreased risk for Alzheimer's disease	Link	Link	
1.5	rs4149274	(C;C)	Associated with higher HDL (good) cholesterol.	Link		
1.5	rs4939883	(C;T)	Associated with higher HDL cholesterol	Link	Link	
1.2	rs11172113	(C;C)	0.8x lower risk for migraines	Link		
1.2	rs11246226	(A;C)	Decreased risk of schizophrenia in limited stud...	Link	Link	
1.2	rs6048	(G;G)	Slightly lower risk (10-20%) of deep vein throm...	Link	Link	Link
1.2	rs9306160	(C;T)	0.75x (reduced) risk for metastasis in LN-/ER+ ...	Link	Link	
1.1	rs2293347	(G;G)	Among NSCLC patients: better Gefitinib response...	Link		Link
1	rs10248420	(A;G)	7x more likely to respond to certain antidepres...	Link	Link	
1	rs11983225	(C;T)	7x more likely to respond to certain antidepres...	Link	Link	
1	rs182549	(C;T)	Can digest milk.	Link		Link
1	rs2235040	(A;G)	7x more likely to respond to certain antidepres...	Link	Link	
1	rs2235067	(A;G)	7x more likely to respond to certain antidepres...	Link		
1	rs2494732	(T;T)	Lower odds of psychosis	Link	Link	
1	rs25640	(A;A)	Benign polymorphism	Link	Link	Link
1	rs2952768	(C;T)	Slightly less drug dependence: decreased effect...	Link		Link
1	rs3755319	(G;T)	Most likely a benign polymorphism	Link		Link
1	rs4129267	(C;C)	No increased risk of higher IL-R and CRP levels...	Link	Link	
1	rs4148739	(A;G)	7x more likely to respond to certain antidepres...	Link	Link	
1	rs4939827	(C;T)	0.86x decreased risk for colorectal cancer	Link	Link	Link
1	rs7850258	(A;G)	Typical odds of developing primary hypothyroidi...	Link		
1	rs800292	(C;T)	1% decreased risk of macular degeneration	Link	Link	Link
1	rs8179183	(C;G)	Less likely to gain weight if taking risperidon...		Link	
0.1	rs1538660	(C;T)	Likely to be a benign variant	Link	Link	Link
0.1	rs3204145	(A;T)	Likely to be a benign variant	Link	Link	Link

3.2 Possibly Harmful Traits

Mag.	Identifier	Genotype	Summary	GnomAD	GetEvidence	ClinVar
3	rs2066847	(-;C)	3x higher risk of Crohn's disease	Link		Link
3	rs2981582	(C;T)	1.3x higher risk of ER+ breast cancer	Link	Link	
2.7	rs10830963	(C;G)	Increased type-2 diabetes risk; higher gestatio...	Link	Link	
2.5	rs10974944	(C;G)	Increased odds (2 - 4 fold?) of V617F-associate...	Link	Link	
2.5	rs12340895	(C;G)	Increased odds (2 fold?) of developing V617F-po...	Link		
2.5	rs12343867	(C;T)	Increased odds (2 fold?) of V617F-associated MP...	Link		
2.5	rs12803066	(A;G)	Increased risk of myopia	Link		
2.5	rs13266634	(C;T)	Increased risk for type-2 diabetes	Link	Link	Link
2.5	rs1421085	(C;T)	~1.3x increased obesity risk	Link	Link	Link
2.5	rs16969968	(A;G)	Slightly higher risk for nicotine dependence: l...	Link	Link	Link
2.5	rs2004640	(T;T)	1.4x increased risk for SLE	Link	Link	Link
2.5	rs2254958	(C;C)	1.61x increased risk for Alzheimer's	Link		
2.5	rs3738919	(C;C)	1.94x risk of developing rheumatoid arthritis	Link		
2.5	rs3780374	(A;G)	Substantially increased odds of developing V617...	Link		
2.5	rs4495487	(C;T)	Increased odds (2 fold?) of developing V617F-as...	Link		
2.5	rs5888	(C;T)	3x higher risk for age-related macular degenera...	Link		
2.5	rs664143	(T;T)	Higher risk for number of cancers	Link		
2.5	rs8034191	(C;T)	1.27x lung cancer risk	Link	Link	
2.3	rs1859962	(G;G)	1.28x increased risk for prostate cancer	Link	Link	
2.3	rs7966230	(C;G)	Slightly lower levels of plasma VWF	Link		
2.1	rs10427255	(C;C)	Highest odds of photic sneeze reflex	Link		
2.1	rs10811661	(T;T)	1.2x increased risk for type-2 diabetes	Link	Link	
2.1	rs17070145	(C;C)	Reduced memory abilities	Link		Link
2.1	rs17563	(C;C)	Risk for otosclerosis	Link	Link	Link
2.1	rs2306402	(C;C)	1.18x increased risk for late-onset Alzheimer's...	Link		
2.1	rs4430796	(A;A)	1.38x increased risk for prostate cancer	Link	Link	
2.1	rs4444903	(G;G)	3.5x risk of hep-cancer in cirrhosis patients; ...	Link		Link
2.1	rs646776	(A;A)	1.2x risk of coronary artery disease	Link	Link	
2.1	rs795484	(A;G)	Increased morphine dose requirement and postope...	Link		
2.1	rs944289	(C;T)	1.3x increased thyroid cancer risk	Link	Link	
2	rs10086908	(C;T)	1.7x increased risk for prostate cancer	Link		
2	rs1045642	(C;T)	Slower metaboliser for some drugs	Link	Link	Link
2	rs10488631	(C;T)	2x increased risk of developing SLE; 1.6x incre...	Link	Link	
2	rs1050631	(C;T)	Mean Survival Time of 25 months for esophageal ...	Link		
2	rs1051730	(C;T)	1.3x increased risk of lung cancer	Link	Link	Link
2	rs10871777	(A;G)	Adults likely to be 0.22 BMI units higher	Link		
2	rs10889677	(A;C)	1.5x increased risk for certain autoimmune dise...	Link	Link	
2	rs10984447	(A;A)	>1.17x increased risk for multiple sclerosis	Link	Link	
2	rs11045585	(A;G)	63% chance (higher than average) of docetaxel-i...	Link	Link	
2	rs11123857	(G;G)	2.88-fold risk of bipolar disorder or major dep...	Link		
2	rs11190870	(C;T)	Possibly increased risk of scoliosis	Link		
2	rs1143699	(C;C)	In men: 2.19x risk of type 2 diabetes	Link		
2	rs11572080	(A;A)	>1.81x risk of GI bleeding with NSAID drugs	Link	Link	Link
2	rs1160312	(A;G)	1.6x increased risk of Male Pattern Baldness.	Link	Link	
2	rs1219648	(A;G)	1.20x risk for breast cancer	Link	Link	
2	rs12567232	(A;G)	Increased risk for Crohn's Disease	Link	Link	
2	rs12696304	(C;G)	Prone to aging faster: at least in European pop...	Link		
2	rs13254738	(A;C)	1.18x prostate cancer risk	Link	Link	
2	rs1333048	(A;C)	1.3x increased coronary artery disease risk	Link		
2	rs13376333	(T;T)	~2x higher risk of atrial fibrillation	Link	Link	
2	rs1360780	(C;T)	1.3x increased risk for depression	Link	Link	Link
2	rs144848	(G;G)	Very slightly increased breast cancer risk	Link	Link	Link
2	rs1585215	(A;G)	2x increased risk for Hodgkin lymphoma	Link		

Mag.	Identifier	Genotype	Summary	GnomAD	GetEvidence	ClinVar
2	rs1691053	(A;G)	Increased risk of developing prostate cancer	Link		
2	rs16942	(A;G)	Very slightly increased breast cancer risk	Link	Link	Link
2	rs16944	(G;G)	Slightly increased (~2x or less) risk for certa...	Link	Link	
2	rs1734791	(A;A)	1.4x increased risk for lupus	Link		
2	rs17782313	(C;T)	Adults likely to be 0.22 BMI units higher	Link	Link	Link
2	rs1799853	(T;T)	~ 40% reduction in warfarin metabolism: greater...	Link	Link	Link
2	rs2073963	(G;T)	Increased risk of baldness	Link		
2	rs2143340	(C;T)	Increased risk of dyslexia and poor reading per...	Link		
2	rs2201841	(C;T)	1.5x increased risk for Crohn's disease; 2x inc...	Link	Link	
2	rs2230201	(G;G)	>1.4x risk of lupus	Link		Link
2	rs2274223	(A;G)	1.5x increased risk for stomach and esophageal ...	Link	Link	Link
2	rs2383206	(A;G)	1.4x increased risk for heart disease	Link		
2	rs2383207	(A;G)	Increased risk for heart disease	Link		
2	rs241448	(C;T)	1.51x increased risk for Alzheimer's	Link		Link
2	rs2420946	(C;T)	1.20x risk for breast cancer	Link		
2	rs25487	(G;G)	2x higher risk for skin cancer; possibly other ...	Link	Link	Link
2	rs2707466	(G;G)	Weaker bones	Link	Link	
2	rs2736990	(C;C)	Increased risk of developing Parkinson's Diseas...	Link	Link	
2	rs27388	(A;A)	Increased risk of developing schizophrenia	Link		
2	rs2908004	(C;C)	Weaker bones	Link	Link	
2	rs3212227	(A;C)	Significantly increased risk of developing cerv...	Link		Link
2	rs358806	(C;C)	1.78x increased risk of developing Type-2 diabe...	Link	Link	
2	rs3775948	(G;G)	Slightly higher risk for gout	Link		
2	rs4027132	(A;A)	1.51x increased risk of developing bipolar diso...	Link		
2	rs4129148	(C;G)	3x risk of schizophrenia.	Link	Link	
2	rs4633	(T;T)	Higher risk for endometrial cancer	Link	Link	Link
2	rs4792311	(A;G)	Increased risk of prostate cancer	Link	Link	Link
2	rs493258	(G;G)	1.15x risk of Age Related Macular Degeneration	Link		
2	rs4968451	(A;C)	1.61x increased risk for meningioma	Link		
2	rs520354	(A;A)	Increased risk in men for biliary conditions	Link		
2	rs5759167	(T;T)	Higher prostate cancer risk	Link	Link	
2	rs6441286	(G;T)	1.54x chance of developing primary biliary cirr...	Link	Link	
2	rs6457617	(C;T)	2.3x risk of rheumatoid arthritis	Link	Link	
2	rs6603272	(G;T)	2.74x increased risk of developing schizophreni...	Link		
2	rs6896702	(T;T)	Increased risk of developing Parkinson's Diseas...	Link		
2	rs6922269	(A;A)	1.6x risk of coronary artery disease	Link	Link	
2	rs6997709	(G;T)	1.2x higher risk for hypertension	Link		
2	rs699	(C;T)	Increased risk of hypertension	Link	Link	Link
2	rs7216389	(T;T)	1.5x increased risk for Childhood Asthma.	Link	Link	
2	rs7442295	(A;A)	~4x higher risk for hyperuracemia	Link	Link	Link
2	rs744373	(C;T)	1.17x risk of Alzheimer's	Link		
2	rs7536563	(A;A)	>1.12x risk of multiple sclerosis	Link	Link	
2	rs7794745	(A;T)	Slightly increased risk for autism	Link	Link	Link
2	rs7807268	(C;G)	1.3x risk for Crohn's disease	Link	Link	
2	rs7961152	(A;C)	1.2x higher risk for hypertension	Link		
2	rs828907	(G;T)	Slightly increased risk of bladder cancer and 2...	Link		
2	rs9652490	(A;A)	~2x increased risk for Parkinson's disease: and...	Link	Link	
2	rs965513	(A;G)	1.77x increased thyroid cancer risk	Link	Link	
2.0	rs2156921	(G;G)	1.29x increased risk for depression	Link		
2.0	rs2305795	(A;A)	1.64x higher risk of narcolepsy compared to (G;...	Link		Link
2.0	rs9642880	(T;T)	1.5x increased bladder cancer risk	Link	Link	
1.8	rs6700125	(C;T)	1.2x increased risk for ALS	Link		
1.7	rs8055236	(G;T)	1.9x risk for heart disease	Link	Link	
1.6	rs1537415	(C;G)	1.6x increased risk for periodontitis	Link	Link	
1.6	rs1978237	(C;G)	1.59x risk of Type 2 diabetes	Link		

Mag.	Identifier	Genotype	Summary	GnomAD	GetEvidence	ClinVar
1.6	rs2046210	(T;T)	1.6x increased breast cancer risk in certain wo...	Link	Link	Link
1.6	rs2736100	(G;G)	1.6x higher risk for glioma development	Link	Link	Link
1.6	rs356219	(G;G)	1.6x increased risk for Parkinson's disease	Link		
1.6	rs3764880	(A;A)	1.2 - 1.8x increased tuberculosis risk	Link	Link	
1.5	rs10492519	(A;G)	Slightly increased risk of developing prostate ...	Link		
1.5	rs10757272	(C;T)	1.30x increased risk for Coronary artery diseas...	Link		
1.5	rs10859871	(A;C)	Slight (~1.2x) increase in endometriosis risk	Link		
1.5	rs10883365	(A;G)	1.2x increased risk for developing Crohn's dise...	Link	Link	
1.5	rs12037606	(A;G)	1.22x risk of developing Crohn's disease	Link		
1.5	rs12210050	(C;T)	Slightly higher risk for basal cell carcinoma	Link	Link	
1.5	rs1223271	(A;G)	Slightly increased risk of developing Parkinson...	Link	Link	
1.5	rs12431733	(C;T)	Slightly increased risk of developing Parkinson...	Link	Link	
1.5	rs12498742	(A;A)	1.25 increased risk for gout	Link		
1.5	rs13149290	(C;C)	Slightly increased risk of developing prostate ...	Link		
1.5	rs140701	(A;A)	Increased risk for anxiety disorders	Link		
1.5	rs17221417	(C;G)	1.3x higher risk for Crohn's disease	Link	Link	
1.5	rs1801274	(T;T)	Complex; generally greater risk for cancer prog...	Link	Link	Link
1.5	rs2177369	(C;C)	1.5x increased risk for Alzheimer's disease	Link		
1.5	rs2240340	(A;G)	Slightly increased (1.5x) risk for RA	Link		
1.5	rs2272127	(C;C)	Associated with herpes and schizophrenia	Link		
1.5	rs2280714	(A;A)	1.4x increased risk of SLE	Link		
1.5	rs28694718	(A;A)	>2x higher risk for schizophrenia	Link		
1.5	rs3087243	(A;G)	Increased risk for auto-immune diseases	Link	Link	Link
1.5	rs356220	(T;T)	Increased risk of Parkinson's Disease	Link		
1.5	rs3745516	(A;G)	Slightly increased risk of developing primary b...	Link		
1.5	rs3814570	(C;T)	1.3x increased risk for Crohn's disease with il...	Link		
1.5	rs3825776	(A;G)	1.3x increased risk for ALS	Link	Link	
1.5	rs393152	(A;A)	Increased risk of both PD and AD	Link	Link	
1.5	rs401681	(C;T)	~1.2x increased risk for several types of cance...	Link	Link	
1.5	rs4464148	(C;T)	1.10x increased risk for colorectal cancer	Link		
1.5	rs4538475	(A;G)	Slightly increased risk of developing Parkinson...	Link	Link	
1.5	rs4626664	(A;G)	1.44x increased risk of developing restless leg...	Link	Link	
1.5	rs464049	(T;T)	Increased risk of schizophrenia in limited stud...	Link		
1.5	rs486907	(A;G)	1.5x increased prostate cancer risk	Link	Link	Link
1.5	rs4982731	(C;C)	Possible higher risk of childhood acute lymphob...	Link		
1.5	rs5219	(C;T)	1.3x increased risk for type-2 diabetes	Link	Link	Link
1.5	rs5746059	(A;A)	Slightly higher fat mass	Link		
1.5	rs6435862	(G;T)	1.7x higher risk of aggressive neuroblastoma	Link	Link	
1.5	rs6498169	(A;G)	1.14x risk of multiple sclerosis	Link	Link	
1.5	rs6601764	(C;T)	1.16x increased risk of developing Crohn's dise...	Link	Link	
1.5	rs6656401	(A;G)	1.18x increased risk for late-onset Alzheimer...	Link		
1.5	rs6908425	(C;T)	1.63x increased risk of developing Crohn's dise...	Link	Link	
1.5	rs699473	(C;T)	~1.5x increased brain tumor risk	Link		
1.5	rs7774434	(C;T)	Slightly increased risk of developing primary b...	Link		
1.5	rs9561778	(G;T)	~2x increased risk of adverse drug reactions fr...	Link	Link	
1.5	rs966221	(C;C)	1.5x increased stroke risk certain populations	Link		
1.5	rs995030	(G;G)	Non-protective against testicular cancer	Link	Link	
1.4	rs10134944	(C;T)	1.4x risk of bipolar disorder.	Link	Link	
1.4	rs3131296	(G;G)	1.4x increased risk for schizophrenia	Link	Link	
1.4	rs4795067	(G;G)	Slight increase in risk for psoriatic arthritis...	Link		
1.4	rs6010620	(G;G)	1.4x higher risk for glioma development; but th...	Link	Link	
1.3	rs1042713	(A;G)	1.3x increased risk that pediatric inhaler use ...	Link	Link	Link
1.3	rs10947262	(C;C)	1.3x increased risk for osteoarthritis	Link		
1.3	rs110419	(A;G)	1.3x increased risk for neuroblastoma	Link		
1.3	rs1260326	(C;T)	Slightly higher risk for gout	Link	Link	Link

Mag.	Identifier	Genotype	Summary	GnomAD	GetEvidence	ClinVar
1.3	rs13361189	(C;T)	1.3x increased risk for Crohn's disease	Link	Link	
1.3	rs1375144	(C;T)	1.32x increased risk of developing bipolar diso...	Link		
1.3	rs1434536	(A;G)	1.29x increased breast cancer risk	Link		Link
1.3	rs1746048	(C;C)	1.03 increased risk for coronary heart disease	Link	Link	
1.3	rs2024513	(A;G)	1.3x higher risk for schizophrenia (among Han C...	Link		
1.3	rs34330	(C;T)	1.3x higher risk for endometrial cancer (in Chi...	Link		Link
1.3	rs4712653	(C;T)	Very slightly (~1.3x) increased risk for neurob...	Link		
1.3	rs4958847	(A;G)	1.3x increased risk for Crohn's disease	Link		
1.25	rs13387042	(A;A)	1.24x increased risk for breast cancer	Link	Link	
1.2	rs10865331	(A;G)	1.2x higher risk for ankylosing spondylitis	Link		
1.2	rs1344706	(T;T)	1.2x increased risk for schizophrenia	Link	Link	
1.2	rs143383	(C;T)	1.1x increased risk for osteoarthritis	Link	Link	Link
1.2	rs1800693	(A;G)	Slight (1.2x) increase in risk for multiple scl...	Link	Link	Link
1.2	rs2056116	(A;G)	1.18x risk for breast cancer	Link		
1.2	rs2072590	(G;T)	1.2x increased risk for ovarian cancer	Link		
1.2	rs2076295	(G;T)	One copy of the risk allele (G): slightly incre...	Link		
1.2	rs2252586	(A;G)	1.2x higher risk for glioma development	Link		
1.2	rs6897876	(C;C)	Slight increase in testicular cancer risk for m...	Link		
1.2	rs7514229	(G;G)	Associated with early-onset autoimmune thyroid ...	Link		
1.2	rs8050136	(A;C)	1.2x increased risk for T2D in some populations...	Link	Link	
1.2	rs851715	(A;A)	Risk of nonsense-word repetition problems if sp...	Link		
1.17	rs17465637	(A;C)	1.17x higher risk for myocardial infarction	Link	Link	
1.15	rs748404	(C;T)	Very slightly increased risk (1.15) for lung ca...	Link	Link	
1.1	rs11037909	(C;T)	1.27x type II diabetes risk	Link		
1.1	rs1799966	(A;G)	Very slightly increased breast cancer risk	Link	Link	Link
1.1	rs249954	(C;T)	Potentially increased risk of Breast Cancer	Link		Link
1.1	rs2651899	(A;G)	1.1x higher risk for migraines	Link		
1.1	rs2653349	(G;G)	2-6x increased risk for cluster headaches	Link	Link	
1.1	rs34516635	(G;G)	Less longevity for Ashkenazi Jewish women.	Link		Link
1.1	rs3740878	(A;G)	1.26x type II diabetes risk	Link		Link
1.1	rs3818361	(C;T)	1.15x increased risk for late-onset Alzheimer's...	Link		
1.1	rs4977574	(A;G)	Some studies - but not others - report a slight...	Link	Link	
1.1	rs5030737	(C;T)	Carrier of mannose binding deficiency but of lo...	Link	Link	Link
1.1	rs7412	(C;T)	More likely to gain weight if taking olanzapine...	Link	Link	Link
1.1	rs7531806	(A;G)	Very slightly increased risk of acne occurrence...	Link		
1.1	rs925391	(C;C)	More likely to go bald; common	Link		
1.05	rs2291834	(C;T)	Very slightly higher risk for myocardial infarc...	Link		
1	rs1004819	(C;T)	1.5x risk of Crohn's disease: 1.2 for developin...	Link	Link	
1	rs10761659	(A;G)	1.2x risk of Crohn's disease	Link	Link	
1	rs1417066	(C;T)	Slightly increased risk of osteoarthritis	Link		
1	rs17300539	(G;G)	Increased risk of insulin resistance	Link		
1	rs2273697	(A;G)	Adverse reaction more likely to carbamazepine i...	Link	Link	Link
1	rs2282679	(A;C)	Somewhat lower vitamin D levels	Link		
1	rs2546890	(A;G)	Higher risk of multiple sclerosis	Link		
1	rs3194051	(A;A)	>1.1x risk of type-1 diabetes	Link	Link	Link
1	rs5326	(A;G)	Possible psychiatric risks	Link		
1	rs6932590	(T;T)	1.1x increased risk for schizophrenia	Link	Link	
1	rs6974491	(A;G)	Higher risk of coeliac and/or inflammatory bowe...	Link		
1	rs6976	(C;T)	Slight risk of osteoarthritis	Link		
1	rs7453920	(G;G)	Slight increase in risk for chronic hepatitis B...	Link		
1	rs987525	(A;C)	2.5x increased risk for cleft lip	Link	Link	
0.1	rs11110912	(C;G)	Maybe some quite minor increase in high blood p...	Link		
0.1	rs3095870	(G;G)	1.7x increased risk for SLE (lupus)	Link		
0.1	rs601338	(A;G)	Susceptible to Norovirus infections	Link	Link	Link

3.3 Genosets (Multi-variant Phenotypes)

Magnitude	Identifier	Summary
4	gs144	Male
3.5	gs126	Poor warfarin metabolizer
3.3	gs162	CYP2C9 Poor Metabolizers
3.1	gs122	7x risk of male baldness
3.1	gs191	Impaired NSAID drug metabolism
3	gs241	Lighter green: brown or hazel eye color
3	gs273	Lowest risk (13% of white women) of Atrial Fibr...
2.5	gs155	CYP3A5 non-expressor
2.5	gs157	More stimulated by coffee
2.5	gs259	Homozygous for eye color haplotype #3
2.5	gs281	Part of the 88% of the population claimed not t...
2.5	gs285	Claimed to lose 2.5x as much weight on a low fa...
2.3	gs255	Homozygous eye color haplotype #1
2.1	gs223	One copy of GCH1 variant associated with lower ...
2	gs101	Probably able to digest milk
2	gs140	NAT2 slow metabolizer
2	gs154	NAT2 Slow metabolizer
2	gs173	CYP2D6*10
2	gs221	Autoimmune disorder risk in Europeans
2	gs269	APOE E2/E3
2	gs279	Mild trimethylaminuria
1.7	gs233	Normal pain sensitivity; APS/APS: LPS/APS: and ...
1.5	gs185	The beta blocker metoprolol is effective: with ...
1.5	gs220	HLA-B*1502?
1.5	gs247	Parkinson's Disease Risk
1.2	gs184	Able to taste bitterness.
1	gs182	CYP2D6*39

4 Raw Data

The raw data used to create this report has been assigned the identifier PRJEB17529 in the European Nucleotide Archive (ENA) hosted at the European Bioinformatics Institute (EBI).

These data will not be accessible unless the report is approved. This will happen by default one month after the report is issued, or if the report is approved for immediate release within the one month period. Participants can also withdraw from the study at any time in which case the report and the data will not be released and will be deleted.

If the data has already been released, it can be accessed at: http://www.ebi.ac.uk/ena/data/view/PRJEB17529

5 Report Metadata

Resource	Version	Website
Genome	GRCh37	Link
BWA	0.7.12	Link
SAMtools	1.3	Link
GATK	3.4-46	Link
PLINK	v1.90b3.35	Link
SNPedia	02-Aug-2018	Link
GnomAD	v2.0.2	Link
GetEvidence	10-Aug-2018	Link
ClinVar	10-Aug-2018	Link

Table 5: Analysis Pipeline Versions

Report generated on September 11, 2018.
