# Supplementary figures and images for: Personal Genome Project UK (PGP-UK): a research and citizen science hybrid project in support of personalized medicine
Source: BMC Med Genomics. 2018 Nov 27;11:108. doi: 10.1186/s12920-018-0423-1 (PMC6257975; doi:10.1186/s12920-018-0423-1)

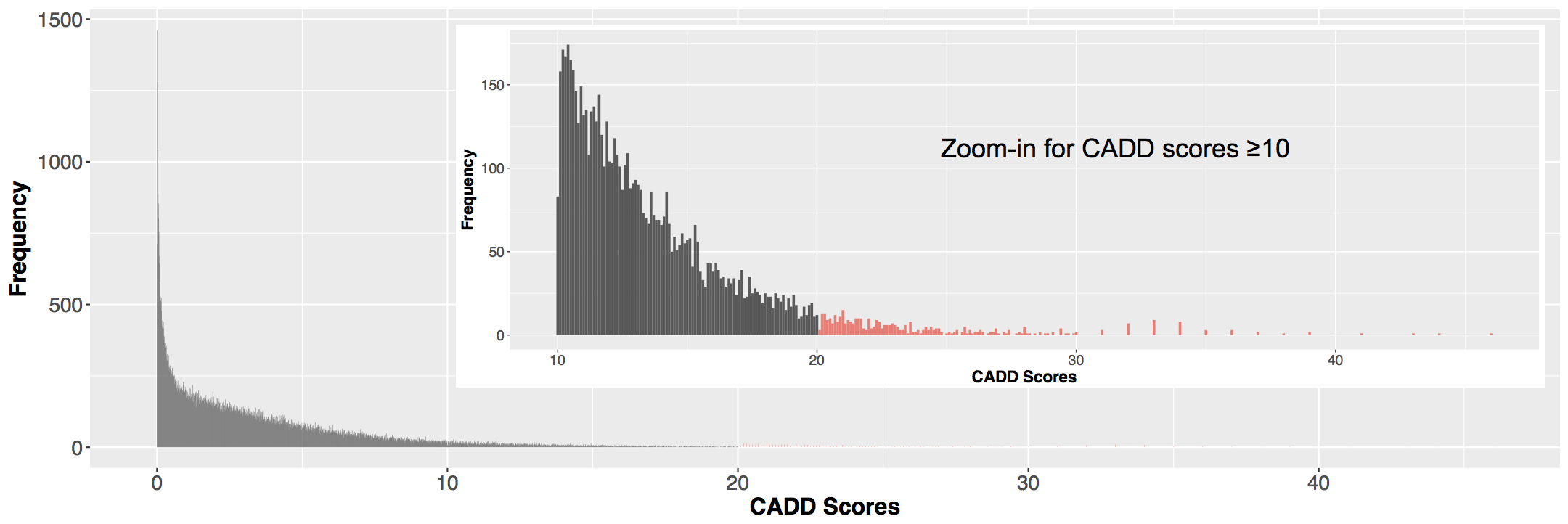

Supplement: Supplementary file 3 — Distribution of private SNVs following effect prediction with multiple methods. SNVs that passed the significance threshold for each method are coloured red. (ZIP 144 kb) [file 12920_2018_423_MOESM3_ESM.zip › AdditionalFile3AR0.png]

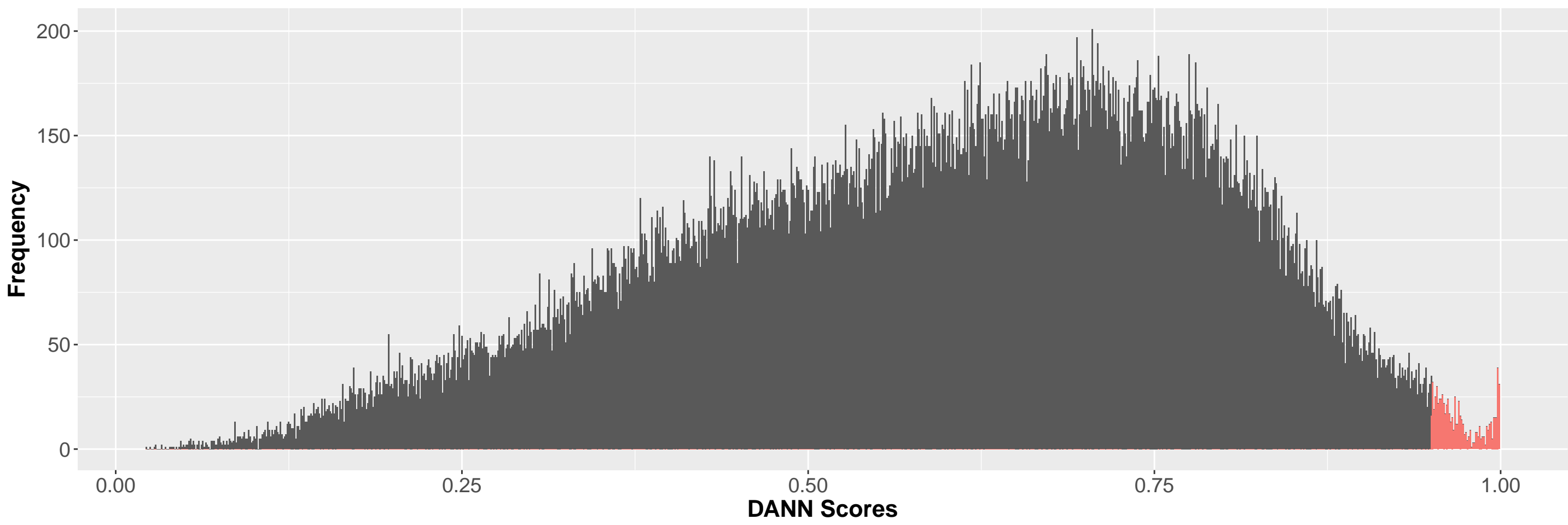

Supplement: Supplementary file 3 — Distribution of private SNVs following effect prediction with multiple methods. SNVs that passed the significance threshold for each method are coloured red. (ZIP 144 kb) [file 12920_2018_423_MOESM3_ESM.zip › AdditionalFile3BR0.pdf]

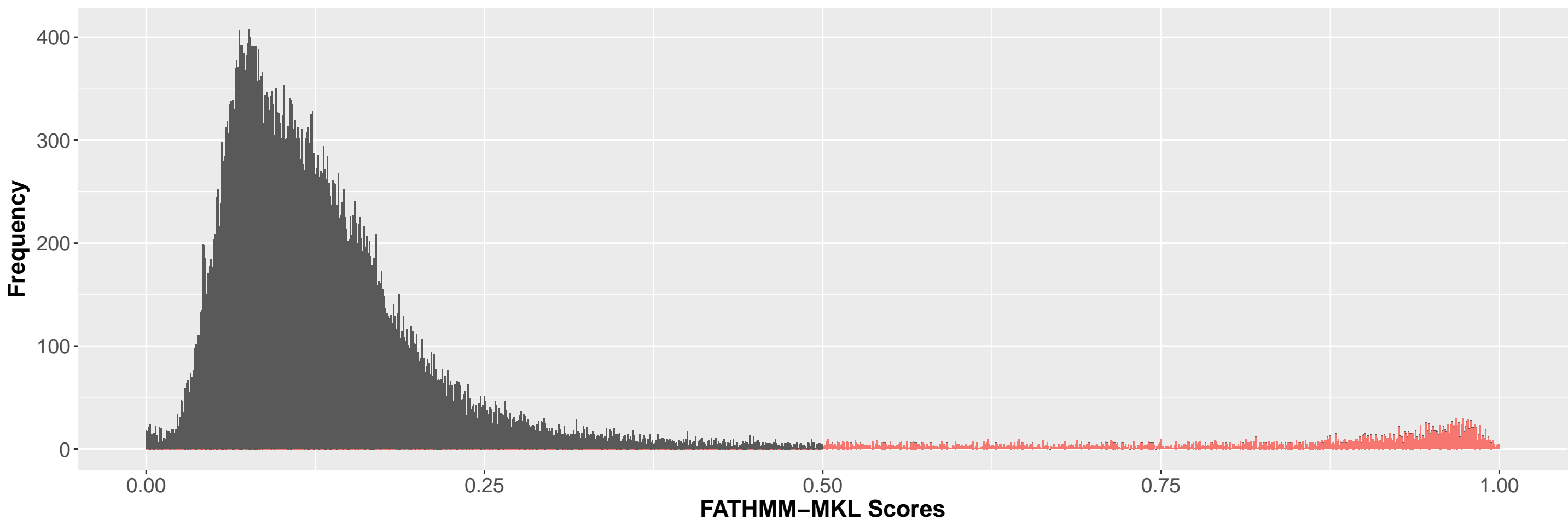

Supplement: Supplementary file 3 — Distribution of private SNVs following effect prediction with multiple methods. SNVs that passed the significance threshold for each method are coloured red. (ZIP 144 kb) [file 12920_2018_423_MOESM3_ESM.zip › AdditionalFile3CR0.pdf]

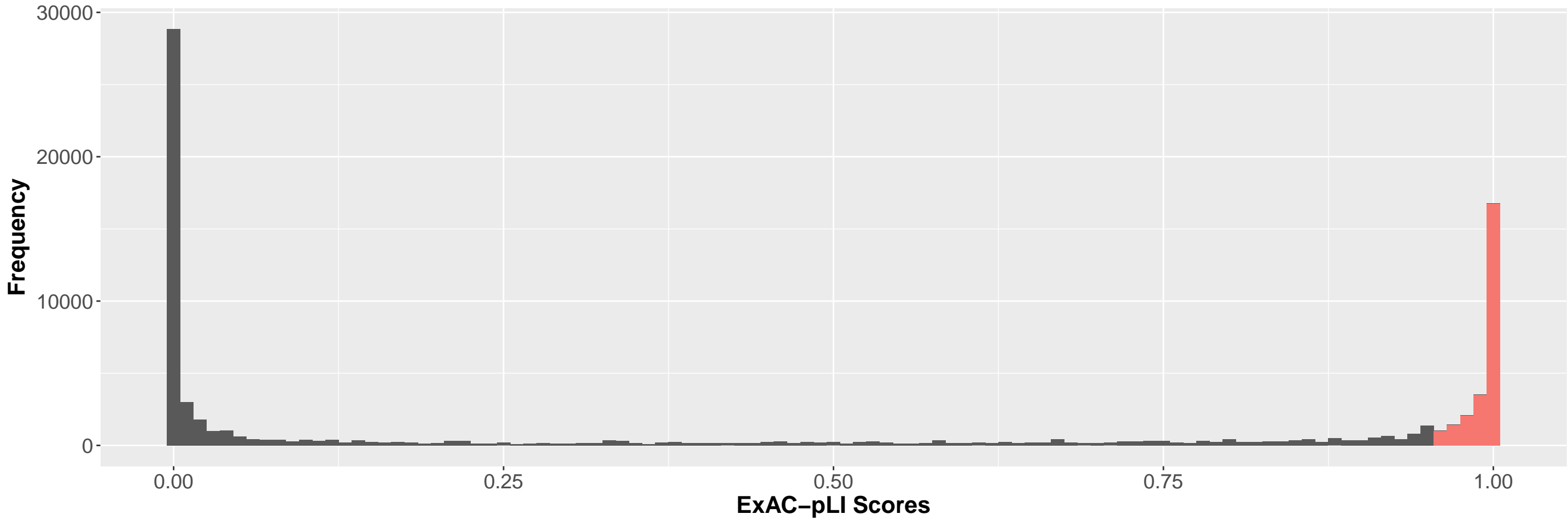

Supplement: Supplementary file 3 — Distribution of private SNVs following effect prediction with multiple methods. SNVs that passed the significance threshold for each method are coloured red. (ZIP 144 kb) [file 12920_2018_423_MOESM3_ESM.zip › AdditionalFile3DR0.pdf]
