# Supplementary material for: Personal Genome Project UK (PGP-UK): a research and citizen science hybrid project in support of personalized medicine
Source: BMC Med Genomics. 2018 Nov 27;11:108. doi: 10.1186/s12920-018-0423-1 (PMC6257975; doi:10.1186/s12920-018-0423-1)
Supplement: Supplementary file 5 — Exemplar PGP-UK 450K methylome report of participant uk35C650 providing predictions for sex, age and smoking status using blood or saliva as indicated. (PDF 223 kb) [file 12920_2018_423_MOESM5_ESM.pdf]

Methylome (450K) Report for uk35C650

1 Summary

Epigenetics is the study of modifications of the DNA which control if a gene is switched on or off, without changing the DNA sequence itself. Epigenetic changes are important in many biological processes in human health and disease. There are several different types of epigenetic modifications, of which DNA methylation is the most studied. DNA methylation involves the addition or removal of a methyl group (CH_3) to/from cytosine bases in the DNA.

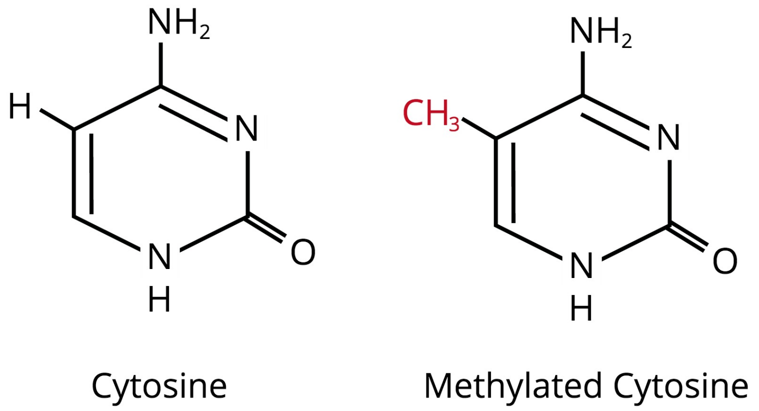

Collectively, all DNA methylation variation within a cell is known as the methylome. The methylome is known to change during normal development, ageing and disease as well as in response to the environment (for example, smoking). It therefore changes throughout life. The methylome is also different in different tissues of the body, such as the brain, skin or blood.

The methylome can be used to predict many features including a person's age, sex and smoking status (current or past/never). DNA methylation differences are widely expected to become biomarkers for environmental exposures, to be used in early diagnosis of disease and to allow matching of patients to the most appropriate disease therapies. As new reliable biomarkers become established they will also be reported for PGP-UK participants.

This report summarises the analysis results of different features from the methylomes of blood and/or saliva. The data were generated using an array-based method from Illumina. The array allows analysis of DNA methylation at around half a million (450K) sites spread across the methylome.

This report was generated automatically and is not clinically approved. It is provided for personal and research purposes only.

2 Prediction of age

A small number of methylation sites in the methylome change throughout a person's lifetime in a predictable way. This allows DNA methylation data to be used to predict a person's current age. By measuring 353 such sites using a methylation array, we have predicted the age of the participant using saliva and/or blood samples. This was carried out using the epigenetic clock [1] which was developed by Steve Horvath at the University of California. If the predicted methylation age deviates from the self-reported actual age at the time of sampling, we further predict age acceleration (where the methylation age is higher than the actual age) and age deceleration (where the methylation age is lower than the actual age). Acceleration and deceleration are shown if the difference is more than 3.6 years (which is the range of accuracy for the epigenetic clock).

Deviations between actual and methylation age can give an insight into general health. Studies have recently associated extreme methylation age acceleration with certain types of cancer [2] and overall mortality [3], while methylation age deceleration has been associated with longevity [4].

PGP Participant	uk35C650

Sample Tissue	Saliva
Predicted Age	53 years, 5 months
Age at Sampling	59 years, 8 months

Sample Tissue	Blood
Predicted Age	52 years, 9 months
Age at Sampling	59 years, 8 months

Sample Tissue	Saliva
Predicted Age	51 years, 4 months
Age at Sampling	59 years, 8 months

Sample Tissue	Blood
Predicted Age	56 years, 4 months
Age at Sampling	61 years, 11 months

3 Prediction of sex

Females have two X chromosomes but only require one of them to be active. The other X chromosome is inactivated by DNA methylation and other silencing mechanisms. By measuring DNA methylation levels on the X chromosome, sex can be predicted [5].

PGP Participant	uk35C650
Self Reported Sex	Male

Sample Tissue	Saliva
Predicted Sex	Male
Fraction X Chromosome Methylation	0.337551761450253

Sample Tissue	Blood
Predicted Sex	Male
Fraction X Chromosome Methylation	0.339751421837792

Sample Tissue	Saliva
Predicted Sex	Male
Fraction X Chromosome Methylation	0.332147528543224

Sample Tissue	Blood
Predicted Sex	Male
Fraction X Chromosome Methylation	0.328610239082086

4 Prediction of exposure to smoking

One of the most well validated exposures which alters DNA methylation is exposure to tobacco smoking. Many studies have shown that DNA methylation at hundreds of sites across the genome changes when someone smokes, particularly at a gene called AHRR. Studies have also found that while previous smokers still have traces of methylation differences, the DNA methylation changes associated with smoking gradually change to be more similar to the methylation of people who have never smoked. A recent study found that these methylation sites change much more in the buccal cells (cells from the epithelial lining of the mouth) of smokers compared to blood cells.

The smoking status for PGP-UK participants was predicted from saliva and/or blood using 187 methylation sites which have been found to change in smokers [6]. Using a method previously described [7], the methylation levels at these sites were used to generate a weighted methylation score, which can be used to differentiate between past/never and current smokers. It has been demonstrated that if a participant has a score of more than 17.55 for Europeans, or more than 11.79 for South Asians, they are classified as current smoker. A limitation of this measure is that the smoking scores have not been tested comprehensively in people of different ethnicities, so we do not yet know the exact threshold to define smoking status in different ethnicities.

PGP Participant	uk35C650
Self Reported Current Smoker	No
Self Reported Past Smoker	Yes

Sample Tissue	Saliva
Smoking Score	1.54594612716571
Smoking Prediction	Past/Never Smoker

Sample Tissue	Blood
Smoking Score	-1.60092756075582
Smoking Prediction	Past/Never Smoker

Sample Tissue	Saliva
Smoking Score	1.49212699345117
Smoking Prediction	Past/Never Smoker

Sample Tissue	Blood
Smoking Score	-1.60989755334765
Smoking Prediction	Past/Never Smoker

5 Appendix

5.1 Methylation sites used in epigenetic age prediction (n=353)

cg00075967	cg00374717	cg00864867	cg00945507	cg01027739	cg01353448	cg01584473
cg01644850	cg01656216	cg01873645	cg01968178	cg02085507	cg02154074	cg02217159
cg02331561	cg02332492	cg02364642	cg02388150	cg02479575	cg02489552	cg02580606
cg02654291	cg02827112	cg02972551	cg03103192	cg03167275	cg03270204	cg03565323
cg03588357	cg03760483	cg04084157	cg04126866	cg04528819	cg04836038	cg05250458
cg05294243	cg05365729	cg05675373	cg05755779	cg05921699	cg05960024	cg06121469
cg06144905	cg06361108	cg06462291	cg06493994	cg06557358	cg06738602	cg06810647
cg06952310	cg06993413	cg07285276	cg07291563	cg07337598	cg07455279	cg07595943
cg08030082	cg08090772	cg08124722	cg08251036	cg08370996	cg08413469	cg08434234
cg08771731	cg08965235	cg09019938	cg09118625	cg09191327	cg09418283	cg09509673
cg09785172	cg09869858	cg09885951	cg10281002	cg10376763	cg10377274	cg10486998
cg10523019	cg10920957	cg11932564	cg12351433	cg12373771	cg12768605	cg12830694
cg12946225	cg13038560	cg13216057	cg13319175	cg13460409	cg13682722	cg13836627
cg13854874	cg13899108	cg13975369	cg14258236	cg14308452	cg14329157	cg14424579
cg14501253	cg14658362	cg14723032	cg14894144	cg14992253	cg15341340	cg15381769
cg15547534	cg15661409	cg15974053	cg15988232	cg16150435	cg16241714	cg16494477
cg16547529	cg16579101	cg17063929	cg17099569	cg17285325	cg17408647	cg17655614
cg17729667	cg17853587	cg17960516	cg18055007	cg18180783	cg18440048	cg18573383
cg18983672	cg18984151	cg19008809	cg19167673	cg19273182	cg19305227	cg19346193
cg19478743	cg19514928	cg19692710	cg19945840	cg20295671	cg20305610	cg20524216
cg20692569	cg20761322	cg20795863	cg20828084	cg20914508	cg20947775	cg20999813
cg21096399	cg21378206	cg21460081	cg21801378	cg21870884	cg22006386	cg22289837
cg22432269	cg22449114	cg22679120	cg22736354	cg22809047	cg22901840	cg22920873
cg23517605	cg23662675	cg23941599	cg24116886	cg24126851	cg24254120	cg24262469
cg24450312	cg24580001	cg24834740	cg25070637	cg25148589	cg25505610	cg25552492
cg25683012	cg25771195	cg25781123	cg26003813	cg26005082	cg26045434	cg26297688
cg26372517	cg26453588	cg26620959	cg26842024	cg26845300	cg27092035	cg27169020
cg27319898	cg27377450	cg27413543	cg27494383	cg00091693	cg00168942	cg00431549
cg00436603	cg01027805	cg01234063	cg01262913	cg01407797	cg01459453	cg01485645
cg01511567	cg01560871	cg01570885	cg01820374	cg02047577	cg02071305	cg02275294
cg02335441	cg03019000	cg03286783	cg03330058	cg03578041	cg03682823	cg03891319
cg03947362	cg04005032	cg04094160	cg04121983	cg04268405	cg04431054	cg04452713
cg04474832	cg04999691	cg05442902	cg05590257	cg05847778	cg05903609	cg06044899
cg06117855	cg06513075	cg06688848	cg06836772	cg06926735	cg07158339	cg07388493
cg07408456	cg07498421	cg07663789	cg07730301	cg07770222	cg07849904	cg08186124
cg08331960	cg09133026	cg09441152	cg09646392	cg09722397	cg09722555	cg09809672
cg10045881	cg10266490	cg10345936	cg10865119	cg10940099	cg11025793	cg11299964
cg11314684	cg11388238	cg11653266	cg12413566	cg12616277	cg12941369	cg12985418
cg13129046	cg13269407	cg13302154	cg13547237	cg13828047	cg13931228	cg14060828
cg14163776	cg14175438	cg14408969	cg14409958	cg14423778	cg14597908	cg14654875
cg14727952	cg15185286	cg15262928	cg15703512	cg15804973	cg16034652	cg16168311
cg16358826	cg16408394	cg16419345	cg16744741	cg16899442	cg16984944	cg17274064
cg17324128	cg17338403	cg17589341	cg17686885	cg18031008	cg18139769	cg18328933
cg18956095	cg19044674	cg19046959	cg19420968	cg19569684	cg19706682	cg19722847
cg19724470	cg19761273	cg19853760	cg20100381	cg20240860	cg21211748	cg21305265
cg21370143	cg21395782	cg21950518	cg22171829	cg22190114	cg22197830	cg22568540
cg22613010	cg22637507	cg22947000	cg23092072	cg23124451	cg23180365	cg23786576
cg24058132	cg24081819	cg24471894	cg24888049	cg24899750	cg25101936	cg25159610
cg25166896	cg25411725	cg25564800	cg25657834	cg25809905	cg25928579	cg26043391
cg26162695	cg26394940	cg26456957	cg26614073	cg26723847	cg26824091	cg27015931
cg27016307	cg27202708	cg27544190				

5.2 Methylation sites used in smoking prediction (n=187)

cg09469355	cg08884752	cg12547807	cg04885881	cg21393163	cg21913886	cg19713429
cg27537125	cg15542713	cg24049493	cg23090529	cg21140898	cg19406367	cg25189904
cg09662411	cg18146737	cg12876356	cg18316974	cg09935388	cg11231349	cg08709672
cg20295214	cg03547355	cg17819085	cg23079012	cg06635952	cg26271591	cg23667432
cg03188382	cg19713851	cg27241845	cg03329539	cg06644428	cg05951221	cg21566642
cg01940273	cg13193840	cg17024919	cg15693572	cg23480021	cg03274391	cg00501876
cg18642234	cg15417641	cg00336149	cg21188533	cg19859270	cg02657160	cg25197194
cg08202836	cg21121843	cg19719391	cg24556382	cg11554391	cg17924476	cg08606254
cg12806681	cg03991871	cg23916896	cg11902777	cg01899089	cg05575921	cg26703534
cg01097768	cg14817490	cg25648203	cg21161138	cg03604011	cg24090911	cg13039251
cg05673882	cg26908328	cg16786458	cg14580211	cg12513616	cg01882991	cg06126421
cg14753356	cg24859433	cg15342087	cg17619755	cg10807309	cg15474579	cg00931843
cg00921574	cg19717773	cg02451831	cg08972170	cg19089201	cg22132788	cg04180046
cg12803068	cg07826859	cg03440944	cg21322436	cg25949550	cg11207515	cg17372101
cg12276019	cg24540678	cg13518625	cg19589396	cg25305703	cg12075928	cg26361535
cg13787850	cg01692968	cg13910681	cg22539182	cg25953130	cg27312979	cg25421530
cg01744331	cg07123182	cg16556677	cg26963277	cg04039799	cg09197783	cg16611234
cg19254163	cg21611682	cg14624207	cg01901332	cg11660018	cg23771366	cg03234777
cg26282236	cg02583484	cg04158018	cg23681440	cg23126342	cg25491122	cg06885459
cg17487894	cg01731783	cg22851561	cg24996979	cg10919522	cg13976502	cg13038618
cg05875421	cg05284742	cg06819357	cg26242531	cg11730703	cg01208318	cg15022400
cg03489965	cg18335991	cg00310412	cg11152412	cg23161492	cg05194346	cg01207684
cg09099830	cg03155159	cg00911794	cg23621097	cg09858022	cg19572487	cg04956244
cg16255816	cg03373393	cg25809905	cg21280392	cg07465627	cg02186444	cg07251887
cg06459104	cg00073090	cg15187398	cg07381806	cg00835193	cg03636183	cg15159987
cg23973524	cg11902728	cg22649124	cg11701312	cg16201146	cg07339236	cg00871610
cg06595162	cg23110422	cg22635096	cg02532700	cg01127300		

6 Raw Data

The raw data used to create this report has been assigned the identifier E-MTAB-5377 in the ArrayExpress Archive hosted at the European Bioinformatics Institute (EBI).

The dataset can be accessed at: https://www.ebi.ac.uk/arrayexpress/experiments/E-MTAB-5377/

7 References

1) https://labs.genetics.ucla.edu/horvath/dnamage/

2) Lin 2015, Epigenetic ageing signatures are coherently modified in cancer (PLoS Genet.) DOI: 10.1371/journal.pgen.1005334

3) Marioni 2015, DNA methylation age of blood predicts all-cause mortality in later life (Genome Biology) DOI: 10.1186/s13059-015-0584-6

4) McEwen 2017, Differential DNA methylation and lymphocyte proportions in a Costa Rican high longevity region (Epigenetics & Chromatin) DOI: 10.1186/s13072-017-0128-2

5) Fortin 2014, Minfi Tutorial BioC2014 (Bioconductor)
https://www.bioconductor.org/help/course-materials/2014/BioC2014/minfi_BioC2014.pdf

6) Zeilinger 2013, Tobacco smoking leads to extensive genome-wide changes in DNA methylation (Plos One) DOI: 10.1371/journal.pone.0063812

7) Elliott 2014, Differences in smoking associated DNA methylation patterns in South Asians and Europeans (Clin Epigenetics) DOI: 10.1186/1868-7083-6-4

Report generated on February 14, 2018.
